# Assessment of adherence to acute pulmonary embolism management guidelines: a single-centre registry

**DOI:** 10.1186/s43044-026-00771-2

**Published:** 2026-07-25

**Authors:** Hesham Salah Eldin Taha, Noha Hassanein, Heba Mostafa Kamal, Mirna Mamdouh Shaker, Mohamed Osama, Nouran Hisham

**Affiliations:** 1 Department of Cardiovascular Medicine, Cairo University, Cairo, Egypt; 2 National Heart Institute, Giza, Egypt

**Keywords:** Pulmonary embolism, Guideline adherence, Risk stratification, Socioeconomic disparities

## Abstract

**Background:**

Pulmonary embolism (PE) remains a complex clinical challenge with significant morbidity and mortality. Despite significant advancements in diagnosis and management, as well as the availability of recent guidelines, disparities in their implementation persist due to socioeconomic factors and other barriers. The study aims to assess the degree of implementation of guidelines in the diagnosis and management of PE.

**Results:**

Among 8,648 patients presenting to the National Heart Institute emergency department, 200 were diagnosed with acute PE, accounting for 2.3% of ED visits during the study period. The mean age was 56.7 ± 9.8 years, with 58% being female. Dyspnea was the most common symptom, occurring in 64% of cases, while bed rest for > 3 days was the most frequent risk factor (32%). Computed tomography pulmonary angiography (CTPA) confirmed PE in 95.5% of cases. Risk stratification classified 80% as low to intermediate-low risk, 13% as intermediate-high risk, and 6% as high-risk. Unfractionated heparin monotherapy was initiated in 63% of patients, while only 8.5% were discharged on direct oral anticoagulants (DOACs). Mortality was 3%, including 1% due to life-threatening bleeding. There was notable overuse of D-dimer testing, underutilization of ventilation–perfusion (V/Q) scans, limited prescription of DOACs, and absence of interventional therapies in eligible patients.

**Conclusions:**

This study highlights critical gaps in adherence to PE management guidelines, influenced by physician discretion, socioeconomic constraints, and diagnostic inconsistencies. Strengthening outpatient care pathways, refining risk stratification practices, and ensuring consistent guideline implementation are key priorities to optimize outcomes.

## Background

Pulmonary Embolism (PE) remains a major health problem, ranking as the third leading cause of cardiovascular-related deaths, following myocardial infarction (MI) and stroke. However, clinical presentations of PE vary widely across patients ranging from incidental asymptomatic PE to fatal PE [1].

High clinical suspicion is needed while seeing a patient with cardiopulmonary symptoms. Risk stratification with clinical scores, biomarkers and imaging help to refine the best treatment strategy. The goal of PE treatment is to reduce the risk of the acute and long-term complications, including mortality and recurrent PE [2].

The current guidelines recommend risk stratification of patients with PE at initial diagnosis, as it helps in therapeutic decision-making in the acute phase, including the identification of high-risk patients, who might benefit from more aggressive therapy such as thrombolysis, embolectomy and mechanical support [2].

In the current study, we aimed to assess the level of adherence to clinical practice guideline recommendations in managing patients presenting with acute PE.

## Methods

This was an observational cross-sectional study to assess the degree of implementation of guidelines in acute PE management. The management was solely determined by the treating physician to identify practices for the management of patients and related outcomes.

The study was conducted at the National Heart Institute, Giza, Egypt, throughout the period from March 2023 to April 2024. The study protocol was registered and approved by the local research ethics committee (MD-52-2023).

### Study Population:

Patients suspected of having a PE were assessed based on demographics, comorbidities, risk factors, presentation modes, investigative findings, management and outcomes during their hospital stay.

### Inclusion criteria:

Adults aged 18 years or older with a confirmed diagnosis of acute PE, including both first-time and recurrent episodes, were eligible for inclusion.

### Exclusion criteria:

Patients with suspected PE who experienced cardiac arrest before confirmation of the diagnosis or those who declined participation in the study were excluded.

### Study Workup:

Following a written informed consent, recruited patients underwent evaluation to ensure they met the eligibility criteria. The index event was defined as symptomatic PE, with or without deep venous thrombosis (DVT), diagnosed in the emergency department (ED) and confirmed through tests like transthoracic echocardiography (TTE), computed tomography pulmonary angiography (CTPA), or lower limb (LL) venous duplex ultrasonography.

During the index event, a comprehensive set of variables was recorded, including clinical data such as presenting symptoms, vital signs, and electrocardiographic (ECG) findings. Laboratory investigations captured levels of D-dimer, troponin, international normalized ratio (INR), hemoglobin, platelets, and serum creatinine. Additionally, patient information encompassed demographic characteristics, pre-existing comorbidities, and risk factors for venous thromboembolism (VTE).

The Pulmonary Embolism Severity Index (PESI) and simplified PESI (sPESI) scores were employed for prognostication, with patient stratification guided by European Society of Cardiology (ESC) criteria. Risk groups were determined based on clinical scores, evidence of right ventricular (RV) dysfunction, and markers of myocardial injury. High-risk patients were those presenting with shock or hypotension. Intermediate-high-risk patients had PESI class III–V or sPESI ≥ 1, along with RV dysfunction (identified on transthoracic echocardiography or computed tomography pulmonary angiography) and elevated troponin levels. Intermediate-low-risk patients met the same score thresholds but exhibited either RV dysfunction or elevated troponin, not both. Patients lacking all these findings were categorized as low risk.

Documented management strategies included the use of anticoagulants, systemic thrombolysis, vasopressors, inotropes, mechanical ventilation, mechanical circulatory support, surgical embolectomy, percutaneous catheter-directed interventions, and inferior vena cava (IVC) filters. Additional data included length of hospital stay and clinical outcomes, such as mortality and bleeding events.

### Definition of guideline adherence

Guideline adherence in this study was assessed with respect to the 2019 ESC guidelines for acute PE. We focused on key diagnostic and management steps that are explicitly recommended and could be reliably captured in the registry: appropriate use of D-dimer according to pre-test probability, performance of CTPA in eligible patients, use of systemic thrombolysis in high-risk or deteriorating patients, provision of mechanical ventilation when indicated, prescription of anticoagulation at discharge, and application of Hestia criteria for outpatient management. For each of these items, adherence was calculated as the proportion of eligible patients managed in accordance with the guideline recommendation. We did not construct a single composite adherence score, and recommendations were not differentially weighted; instead, adherence is reported parameter-wise.

### Statistical analysis

Statistical analysis was conducted using IBM SPSS for Windows version 26. Categorical data were represented as frequency and percentages, while continuous data were represented as either mean ±standard deviation (SD), or median [inter-quartile range (IQR)] according to normality of values distribution. In-between groups, comparisons were conducted for categorical data by Chi-square or Fischer exact test as appropriate, while for continuous data by independent sample *t*-test or by the Mann-Whitney as appropriate. Repeated measures were contrasted either by Mc-Nemar or repeated measures using ANOVA. A *p*-value of 0.05 was considered the threshold for statistical significance.

Given the descriptive design and sample size, multivariable regression was not performed. Instead, qualitative patterns associated with non-adherence were explored descriptively.

## Results

**Fig. 1:**
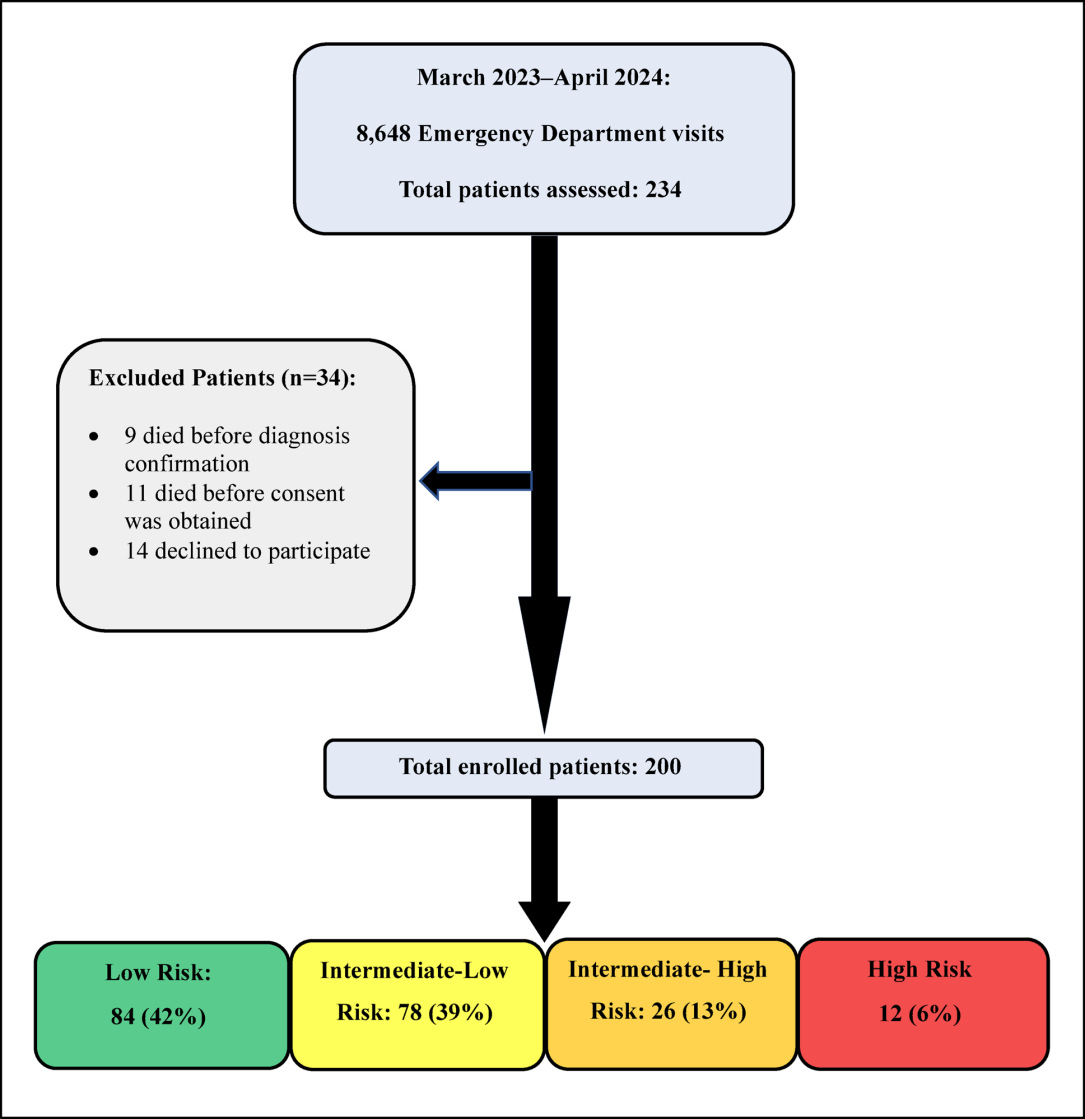
Flowchart of enrolled patients

A total of 234 patients aged 18 years or more were assessed for eligibility. Of these, 200 patients were successfully enrolled between March 2023 and April 2024. A total of 34 patients were excluded for the following reasons: 9 patients passed away prior to the confirmation of diagnosis, 11 patients died before informed consent could be obtained, and 14 patients declined participation in the study (Fig. 1). The incidence of PE during this period was 200 out of 8648 ED visits, accounting for 2.3% of the total ED visits.

In the study population, the mean age was 56.7 years, with women comprising 58% of participants. The average body mass index (BMI) was 25.8 kg/m², and 26% of the patients were smokers. The most common presenting symptom at the time of diagnosis was dyspnea, reported in 64.5% of cases, followed by chest pain in 16%. Only 1% of patients presented with cardiac arrest and required resuscitation (Table 1).

**Table 1:** Patient demographic criteria and modes of presentation

Variables	Number (%) or mean *±* SD (*n* = 200)
Patient Characteristics	
Age (years)	56.7 ± 9.8
Gender (female)	116 (58%)
Current smokers	52 (26%)
BMI (kg/m^2^)	25.8 ± 4.2
Systolic BP (mmHg)	119.95 ± 13.8
Diastolic BP (mmHg)	76.16 ± 8.75
Heart rate (bpm)	101.38 ± 25.96
Oxygen saturation (%)	88.45 ± 6.1
Mode of presentation	
Dyspnea	129 (64.5%)
Chest pain	32 (16%)
Syncope	3 (1.5%)
Hemoptysis	24 (12%)
Shock	8 (4%)
Cardiac arrest	2 (1%)

*BMI* Body Mass Index; *BP* Blood Pressure; *BPM* Beat per minute

Regarding risk factors of PE, the most common was bed rest exceeding three days, reported in 32% of patients. This was followed by a history of previous venous thromboembolism (VTE) in 24%, and cancer in 18% of cases. Notably, only 7% of patients experienced unprovoked PE (Table 2). Risk factors for pulmonary embolism in Table 2 are grouped according to the 2019 ESC classification into strong, moderate, and weak predisposing factors for venous thromboembolism.

**Table 2:** Risk factors for pulmonary embolism in study participants*

Variables	Number (%)
Strong risk factors	
Fracture of LL	14 (7%)
Hospitalization for HF or Atrial fibrillation /flutter (within past 3 months	13 (6.5%)
Hip or knee replacement	11 (5.5%)
Major trauma	22 (11%)
Myocardial infarction (within past 3 months)	3 (1.5%)
Previous VTE	48 (24%)
Spinal cord injury	2 (1%)
Moderate risk factors	
Arthroscopic knee surgery	6 (3%)
Autoimmune diseases	15 (7.5%)
Blood transfusion	2 (1%)
Central venous lines	3 (1.5%)
Intravenous catheters and leads	14 (7%)
Chemotherapy	22 (11%)
Congestive heart failure or respiratory failure	0 (0%)
Erythropoiesis-stimulating agents	0 (0%)
Hormone replacement therapy	2 (1%)
In vitro fertilization	1 (0.5%)
Oral contraceptive therapy	7 (3.5%)
Postpartum period	3 (1.5%)
Infection	10 (5%)
Inflammatory bowel disease	2 (1%)
Cancer	36 (18%)
Paralytic stroke	2 (1%)
Superficial vein thrombosis	0 (0%)
Thrombophilia	2 (1%)
Weak risk factors	
Bed rest > 3 days	64 (32%)
Diabetes mellitus	33 (16.5%)
Arterial Hypertension	42 (21%)
Immobility due to sitting	9 (4.5%)
Old age	45 (22%)
Laparoscopic surgery	2 (1%)
Obesity	41 (20.5%)
Pregnancy	2 (1%)
Varicose veins	6 (3%)
Unprovoked PE	14 (7%)

* Classification of risk factors is based on the 2019 ESC pulmonary embolism guidelines

*LL* Lower Limbs; *HF* Heart Failure; *Afib* Atrial Fibrillation; *VTE* Venous Thromboembolism

Clinical probability based on the Geneva score classified 21.5% of patients as low-risk pre-test probability for PE, 54% as intermediate-risk, and 24.5% as high-risk.

Laboratory investigations demonstrated that 94.5% of patients had a positive D-dimer, while 37% had elevated troponin levels. According to guidelines, D-dimer testing was indicated for 151 patients (75.5%) with low and intermediate pre-test probability (PTP) for PE, but it was performed in 189 patients (94.5%), leading to overuse in 38 patients (19%). Meanwhile, 11 high-risk patients (PTP for PE) did not undergo D-dimer testing as per protocol. Overall, 162 patients (81%) were managed in accordance with guideline recommendations.

ECG was appropriately performed in all patients, in accordance with guideline recommendations. ECG findings revealed that sinus tachycardia was the most frequent abnormality, present in 40.5% of patients. Normal sinus rhythm was observed in 34.5% of cases, whereas atrial fibrillation and the S1Q3T3 pattern were each found in 8.5% of patients. Atrial flutter was the least common finding, seen in only 1.5% of cases.

Imaging findings demonstrated that TTE was performed in all patients. Although not mandated for all hemodynamically stable cases, this approach is consistent with the ESC 2019 Class IIa recommendation that RV assessment by imaging should be considered even in patients with low PESI or negative sPESI. TTE revealed right ventricular (RV) dilation in 61% of cases, with the 60/60 sign being the most prevalent echocardiographic abnormality (78%). A positive McConnell sign was observed in 28% of patients, while flattening of the interventricular septum was seen in 21.5%. Additional findings included a distended IVC in 19%, reduced tricuspid annular plane systolic excursion (TAPSE < 17 mm) in 14.5%, and decreased peak systolic velocity of the tricuspid annulus (< 9.5 cm/sec) in 13.5%. A mobile thrombus in the right heart was detected in 1.5% of patients.

**Fig. 2:**
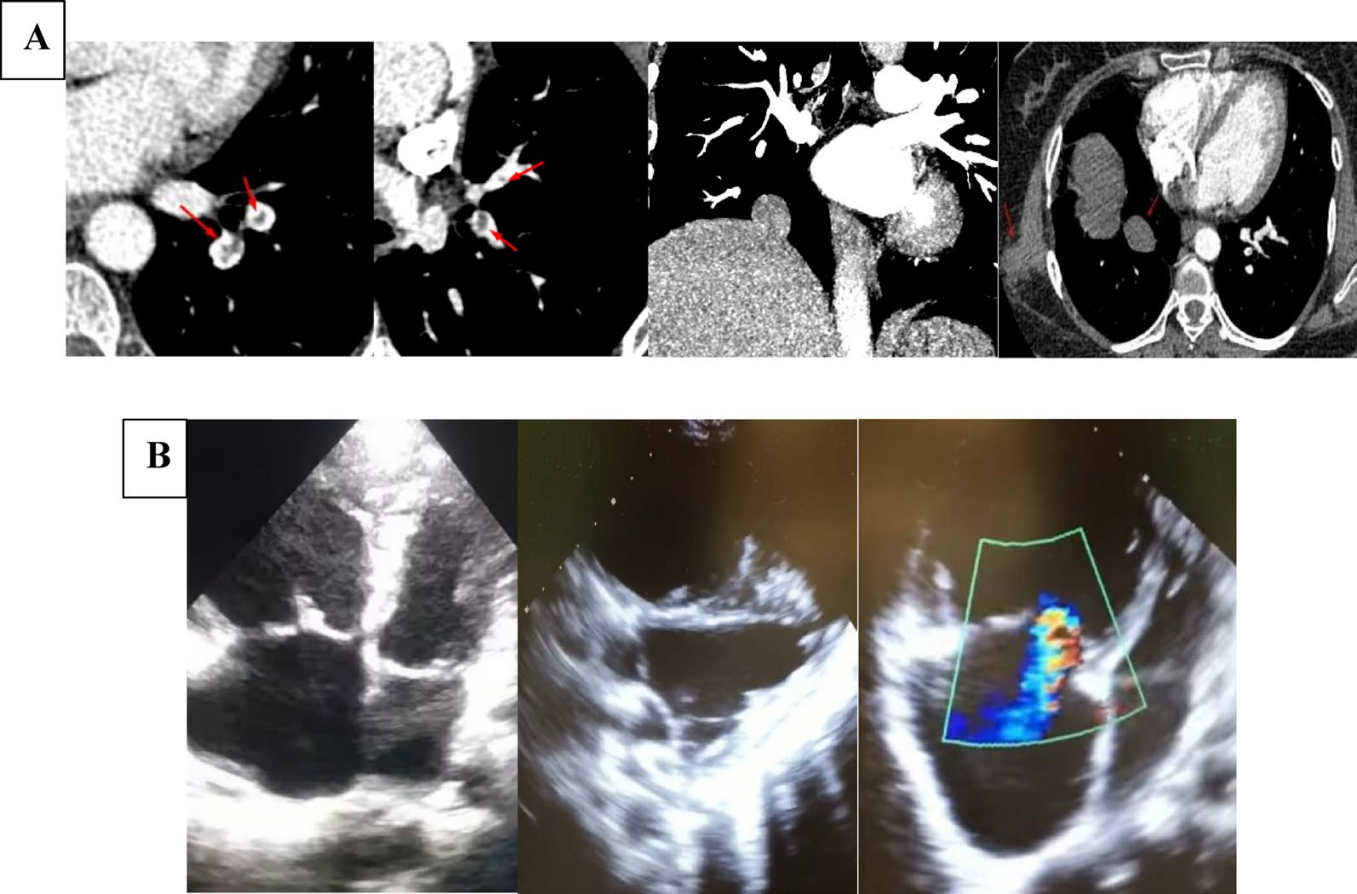
Case of 53-year-old male patient presenting with PE. **A** CTPA showing thrombo-embolism along the lobar branches of both pulmonary arteries, with extension along the segmental branches (on left side). (Red arrows). **B** TTE showing dilated RV and RA with moderate TR and flattened interventricular septum. *PE* Pulmonary Embolism; *CTPA* computed Tomography pulmonary Angiography; *TTE* Transthoracic Echocardiography; *RV* Right Ventricle; *RA* Right Atrium; *TR* Tricuspid Regurgitation

CTPA was performed in 191 of 200 patients (95.5%), confirming pulmonary embolism (PE) in all cases. Segmental arteries were the most involved (58%), followed by lobar arteries (48%). Of the nine patients who did not undergo CTPA, five were ineligible due to renal impairment and four due to hemodynamic instability. In the latter group, diagnosis was established via TTE, and immediate thrombolytic therapy was administered. Figure 2 illustrates imaging findings in a 53-year-old male presenting with pulmonary embolism.

Lower limb venous duplex ultrasonography was conducted in 90% of patients, yielding positive findings in 68.3%, with proximal deep vein thrombosis (DVT) detected in 48.9% of these cases. Laboratory, ECG, and echocardiographic findings are summarized in Table 3.

**Table 3:** Laboratory, electrocardiographic and transthoracic echocardiographic findings in enrolled patients

Investigations	Variables	Number (%)
Laboratory markers	Positive D-dimer	189 (94.5%)
	Positive Troponin (I)	74 (37%)
	INR	1.13 ± 0.25
	Hb (g/dL)	12.64 ± 1.5
	Platelets (10^3^/µL)	307.1 ± 86.1
	Creatinine (mg/dL)	1.05 ± 0.35
ECG	NSR	69 (34.5%)
	Sinus tachycardia > 100 bpm	81 (40.5%)
	AF	17 (8.5%)
	Atrial flutter	3 (1.5%)
	RBBB	9 (4.5%)
	RV strain	4 (2%)
	S1Q3T3	17 (8.5%)
Transthoracic Echocardiography data:	LVEF	63.4 ± 5.67
	Dilated RV	122 (61%)
	Positive McConnell sign	56 (28%)
	Flattened interventricular septum	43 (21.5%)
	Distended IVC	38 (19%)
	60/60 sign	156 (78%)
	Rt heart mobile thrombus	3 (1.5%)
	Decreased TAPSE < 17 mm	41 (20.5%)
	Peak systolic velocity of tricuspid annulus < 9.5 cm/sec	27 (13.5%)
	PASP, mmHg	46.1 ± 10.34
CTPA	Patients underwent CTPA	191 (95.5%)
	Positive CTPA for PA	191 (95.5%)
Affected artery in CTPA	Main PA	32 (16%)
	Rt or Lt pulmonary artery	84 (43%)
	Interlobar artery	62 (32%)
	Lobar artery	92 (48%)
	Segmental arteries	111 (58%)
	Subsegmental arteries	58 (30%)
LL Venous Duplex	Patients underwent LL venous duplex	180 (90%)
	Positive LL venous duplex	123 (68.3%)
Site of DVT	Proximal DVT	88 (48.9%)
	Distal DVT	35 (19.4%)
	No DVT	14 (7.7%)

*INR* International Normalized Ratio; *Hb* Haemoglobin; *NSR* Normal Sinus Rhythm; *AF* Atrial Fibrillation; *RBBB* Right Bundle Branch Block; *RV* Right Ventricle; *LVEF* Left Ventricular Ejection Fraction; *IVC* Inferior Vena Cava; *TAPSE* Tricuspid Annular Plane Systolic Excursion; *PASP* Pulmonary Artery Systolic Pressure; *PA* Pulmonary Artery; *CTPA* Computed Tomography Pulmonary Angiography; *LL* Lower Limb; *DVT* Deep Venous Thrombosis

Risk stratification using the sPESI classified 67.5% of hospitalized patients as higher risk (sPESI ≥ 1), while 32.5% were considered low risk (sPESI = 0) (Table 4). Among the 65 patients with sPESI = 0, right ventricular dilatation was present in 52 patients (80%), while impaired TAPSE (< 17 mm) was observed in 10 patients (15%). These findings demonstrate that a substantial proportion of clinically low-risk patients had evidence of RV dysfunction on imaging, reinforcing the clinical value of routine RV assessment in refining early risk stratification.

Based on ESC 2019 criteria, early mortality risk was predominantly low, with 42% classified as low risk and 39% as intermediate low risk, while only 19% fell into intermediate high- or high-risk categories.

**Table 4:** Pulmonary embolism severity index, simplified version (sPESI) score of studied patients

sPESI score	Number (%)
0 points	65 (32.5%)
≥ 1 point	135 (67.5%)

*PESI* Pulmonary Embolism Severity Index

Ten patients required fibrinolysis due to high-risk presentations or clinical deterioration despite anticoagulation. Among these, 70% (*n* = 7) received thrombolytic therapy immediately after diagnosis, while 30% (*n* = 3) underwent fibrinolysis later the same day, all in accordance with ESC recommendations. Mechanical ventilation was administered to all seven patients with established indications, reflecting 100% adherence to supportive care guidelines.

Regarding anticoagulant therapy during hospitalization, unfractionated heparin was the most used agent (63%), followed by low-molecular-weight heparin (26%). Direct oral anticoagulants were used in only 8.5% of patients (apixaban 4%, rivaroxaban 4.5%), indicating limited adoption of DOAC therapy during the acute phase. Fondaparinux was prescribed in only 2% of cases. Systemic thrombolysis with Streptokinase was administered in 5% of patients. Additional supportive measures included vasopressors in 3.5%, inotropes in 1.5%, and mechanical ventilation in 3.5% of cases (Table 5).

**Table 5:** Treatment strategies of enrolled patients

Variables	Number (%)
Type of prescribed anticoagulant	
Unfractionated Heparin	126 (63%)
LMWH	52 (26%)
Fondaparinux	4 (2%)
Apixaban	8 (4%)
Rivaroxaban	9 (4.5%)
Dabigatran	0 (0%)
Edoxaban	0 (0%)
Thrombolytics	
Streptokinase	10 (5%)
r-tPA	0 (0%)
Urokinase	0 (0%)
Vasopressors	
Norepinephrine	7 (3.5%)
Inotropes	
Dobutamine	3 (1.5%)
Mechanical circulatory support (ECMO)	0 (0%)
Mechanical ventilation	7 (3.5%)
Surgical embolectomy	0 (0%)
Percutaneous catheter-directed treatment	0 (0%)
IVC filter	1 (0.5%)

*LMWH* Low Molecular weight Heparin; *r-tPA* recombinant tissue Plasminogen Activator; *ECMO* Extracorporeal Membrane Oxygenation; *IVC* Inferior Vena Cava

Of the 200 patients enrolled, in-hospital mortality was 3%, corresponding to a survival proportion of 97% (93.6–98.9%). Major bleeding was uncommon, with 99% of patients remaining free of such events (96.4–99.9%). According to the Bleeding Academic Research Consortium (BARC) classification, one patient had hematemesis and a hemoglobin drop of 4.5 g/dL requiring transfusion (BARC type 3 A), and another developed intracerebral hemorrhage following thrombolytic therapy (BARC type 3 C). The mean duration of hospitalization was 2.63 ± 1.37 days.

At discharge, 194 patients were prescribed anticoagulation therapy. Only 9 patients (4.6%) had clear contraindications to DOACs—antiphospholipid syndrome (*n* = 5), pregnancy (*n* = 2), or end-stage renal disease (*n* = 2). Despite this, only 17 (8.5%) were discharged on DOAC. The proportion not receiving a DOAC at discharge was 91.5% (86.7–95.0%) (Table 6).

**Table 6:** In-hospital outcomes and anticoagulation discharge patterns of the patients

In-hospital Outcomes	Number (%) or mean *±* SD (*n* = 200)
Mortality	6 (3%)
Bleeding	2 (1%)
Duration of hospital stay, days	2.63 ± 1.37
Duration of ICU stay, days	2.04 ± 1.1
Planned outpatient anticoagulation without being admitted	11 (5.5%)
Discharge on DOAC	17 (8.5%)
Discharge on VKA	175 (87.5%)
Discharge on enoxaparin	2 (1%)

*ICU* Intensive Care Unit; *DOAC* Direct Oral Anticoagulant; *VKA* Vitamin K Antagonist

Early discharge decisions were also assessed: of the 200 patients, 84 (42%) met the Hestia Criteria for outpatient management. However, only 11 of these patients (13%) were discharged directly from the ED on anticoagulant therapy without hospital admission, highlighting limited implementation of early discharge strategies. The overall degree of adherence to the 2019 ESC guideline recommendations for acute PE management is summarized in Table 7.

**Table 7:** Degree of adherence to guidelines in the diagnosis and management of pulmonary embolism

Parameters	Indicated numbers	Actual numbers	% Degree of implementation
ECG done	200	200	100%
TTE done	200	200	100%
CTPA done	191	191	100%
D-dimer measured	151	189	81%
Use of thrombolytic therapy	10	10	100%
Use of mechanical ventilation	7	7	100%
Discharge on AC therapy	194	194	100%
Planned outpatient AC without admission	84	11	13%

*ECG* Electrocardiogram; *TTE* Transthoracic Echocardiography; *CTPA* Computed Tomography Pulmonary angiography; *AC* Anticoagulant

## Discussion

Despite the presence of well-established recommendations, numerous randomized trials have revealed persistent gaps between guideline-directed management and real-world clinical practice [3, 4]. Bridging this discrepancy is a pressing clinical priority, necessitating evaluation of how evidence-based recommendations are adopted, how practice patterns evolve, and whether these shifts translate into improved patient outcomes. This study aims to assess the real-world implementation of the 2019 ESC guidelines for pulmonary embolism (PE) management within an Egyptian cohort.

Our findings highlight demographic variations in PE prevalence. PE was diagnosed in 2.3% of ED visits in our study, compared to 3% in a large U.S. analysis [5]. Sex-related differences were observed, with 58% of PE cases occurring in women, consistent with rates in Erciyes [6] (63.4%), ICOPER [7] (55%), and PERT [8] (53%). Our cohort had a mean age of 56.7 ± 9.8 years, younger than populations in ESPHERIA [9] (68.6 years), ICOPER [7] (62.3 years), and PERT [8] (61 years), reflecting differences in healthcare access and risk exposure.

Dyspnea was the most common symptom, affecting 64.5% of PE cases in our study, compared to 81.3% in Erciyes [6], 82% in ICOPER [7], and higher rates in women (84.6%) than men (80.9%) in the SERIOUS-PE study [10]. Chest pain varied significantly across studies, occurring in 16% of PE cases in our study but 49% in ICOPER [7], indicating differences in symptom presentation and reporting. Similarly, hemoptysis was observed in 12% of our patients, compared to 7% in ICOPER [7], with the SERIOUS-PE study [10] showing it was less frequent in women (2.4%) than men (5.6%), highlighting sex-related symptom differences.

Among risk factors, our findings reinforce the ESC 2019 guidelines on risk stratification, highlighting immobilization (32%) and previous venous thromboembolism (VTE) (24%) as the most common risk factors, both of which are identified as strong risk factors for predicting PE in the guidelines [2]. These align closely with ICOPER [7] (DVT 49.3%, bed rest 28.1%) and ESPHERIA [9] (immobilization 35.7%, previous PE history 16.1%). Similarly, the RIETE registry [11] identified immobility (22.85%) and cancer (22.56%) among the most common risk factors, with a notable 45.56% of PE cases classified as unknown etiology, emphasizing the challenge of identifying definitive risk factors. The SERIOUS-PE study [10] further emphasized prolonged immobility as a key risk factor in women. Unprovoked PE (7% in our study) was lower than ICOPER [7] (24.9%) and ESPHERIA [9] (35.1%), highlighting differences in risk classification and underlying patient characteristics.

Echocardiographic findings varied across studies. RV dilatation was observed in 61% of our patients, lower than RIETE [11] (89.8%) and Erciyes [6], (65.1%), while ICOPER [7] reported RV hypokinesis in 40%. Impaired TAPSE (< 17 mm) was present in 20.5% of our cases, significantly lower than RIETE [11] (85.7%), likely due to our cohort’s lower-risk profile. McConnell sign (28%), flattened septum (21.5%), and 60/60 sign (78%) in our cohort suggest RV strain, whereas ESPHERIA [9] reported RV dysfunction in only 10.9%. Intracardiac thrombus was found in 1.5% of our cases, less than what has been reported in ICOPER [7] (4%).

RV hypokinesia independently predicted higher 30-day mortality (17%) in PE patients with preserved systemic pressure in ICOPER [7], nearly doubling the risk of death. Despite seeming stable, these patients had a poor prognosis, reinforcing the role of echocardiography in early risk stratification and guiding timely intervention [7].

Although the 2019 ESC guidelines do not mandate echocardiography in all hemodynamically stable patients, our universal use of TTE aligns with the Class IIa recommendation that RV assessment by imaging should be considered even in patients with low PESI or negative sPESI. In our cohort, several clinically low-risk patients (sPESI = 0) demonstrated RV dysfunction—including a high prevalence of RV dilatation—indicating that reliance on clinical scores alone may underestimate early RV strain. These findings reinforce the clinical value of routine RV imaging for refining early risk stratification and differentiating causes of acute dyspnea, even when formal reclassification criteria are not met. However, the prognostic impact of echocardiography was beyond the scope of our study, and our findings should be interpreted as descriptive, raising the potential for detecting occult RV dysfunction even in clinically low-risk patients, rather than as evidence of outcome modification. In contrast, the recently published 2026 AHA/ACC guidelines adopt a more restrictive approach, defining a low-severity Category B subgroup in whom echocardiography is not recommended, a strategy that may risk missing occult RV dysfunction. This divergence highlights evolving international perspectives and explains why our practice remains aligned with ESC guidance but differs from newer U.S. recommendations.

Beyond echocardiography, several diagnostic steps diverged from guideline recommendations. In particular, ventilation–perfusion (V/Q) scanning—specifically recommended for patients with renal impairment—was not utilized. Among the small subset of patients in whom computed tomography pulmonary angiography (CTPA) was contraindicated, diagnosis was made using TTE alone, despite ESC guidance favoring V/Q scanning in this setting. This pattern underscores a reliance on available modalities rather than adherence to guideline-preferred pathways, especially in patients with renal dysfunction.

D-dimer testing was also overused, even in patients with high clinical probability, where imaging should have been prioritized. Although D-dimer is a valuable exclusion tool in low-risk patients, its indiscriminate application may contribute to unnecessary diagnostic steps. Another point worth mentioning was that our focus was on the use of D-dimer as a diagnostic modality, rather than on interpretive adjustments such as age-adjusted thresholds. While such adjustments may have been applied in practice, they were not specifically inquired about or captured in our dataset.

Altogether, these patterns reflect key deviations from guideline-endorsed diagnostic strategies—specifically, the overuse of D-dimer and the underuse of V/Q scanning in renal patients. Addressing these imbalances is critical to ensuring more appropriate, efficient, and individualized PE evaluation [2].

The ICOPER [7] registry reported a 3-month all-cause mortality rate of 17.4%, with 11.4% occurring within the first two weeks, where hemodynamic instability was associated with a higher mortality rate (58.3%) compared to 15.1% in stable patients. In contrast, our study reported an in-hospital mortality rate of 3%. Major bleeding occurred in 10.5% of ICOPER [7] patients, while our study reported a lower major bleeding rate of 1%.

Hospital volume also influenced outcomes. A RIETE substudy [12] found that high-volume hospitals (> 40 cases/year) demonstrated significantly lower mortality rates compared to low-volume centers (< 15 cases/year). Our study reported an in-hospital major bleeding rate of 1%, whereas the RIETE registry documented 30-day rates between 3.3% and 3.9%, increasing with hospital volume. The lower bleeding rate in our study likely reflects the shorter observation period. Mortality in our study was 3%, compared to RIETE’s 5.4% at 30 days (PE-related mortality: 1.7%), suggesting that post-discharge outcomes contributed to the higher RIETE rates. Additionally, the sPESI distribution in RIETE showed a slightly higher proportion of high-risk patients in higher-volume hospitals (71.9%) than in our study (67.5%), which may have influenced differences in complications and mortality [12]. These findings highlight the impact of follow-up duration, hospital experience, and patient risk profiles on reported outcomes.

The PERT Consortium registry [8] showed higher 7-day mortality rates (1.3–16.3%) and 30-day rates (2.6–26.3%) based on risk categories, while our in-hospital mortality rate (3%) aligns with low-risk categories (1.3–2.6%). Our higher proportion of low-risk patients (48% vs. 18.8%) contributed to this difference, whereas PERT’s greater high-risk cases (61.8% vs. 52%) likely explains their increased mortality rates [8].

An important observation concerns therapeutic decision-making. The limited use of DOACs warrants closer scrutiny, as their under-prescription in our setting likely reflects socioeconomic barriers and physician reliance on traditional anticoagulants. Likewise, unfractionated heparin was administered far more often than LMWH, despite guideline recommendations—both the 2019 ESC and 2026 AHA/ACC—favoring LMWH in most patients, including those with severe presentations. Furthermore, streptokinase was the sole thrombolytic agent employed, even though contemporary evidence supports accelerated rtPA regimens for faster reperfusion and reduced risk. These treatment patterns appear to be shaped largely by local practice habits and financial constraints.

Although growing evidence supports the benefits of invasive therapies, including surgical embolectomy and catheter-directed interventions with or without extracorporeal membrane oxygenation (ECMO) for high-risk PE or cases of hemodynamic deterioration, they were not utilized in our study population [2, 13]. This reflects institutional protocols, resource availability, or treatment preferences, highlighting the need for further evaluation of their role in optimizing PE management.

Despite 42% of patients meeting Hestia criteria, only 13% were discharged directly from the ED. This gap likely reflects physician caution, limited outpatient follow-up infrastructure, and socioeconomic barriers such as inadequate home support or concerns about medication affordability, all of which may prompt clinicians to favor inpatient care even when guideline criteria for safe discharge are met. Importantly, no survey was conducted to assess physicians’ familiarity with the Hestia criteria or to explore the specific reasons underlying deviations from guideline-recommended outpatient management.

In contrast to latest ESC recommendations [2], only 17 patients from our study population were discharged on DOACs, despite the absence of clear contraindications in most cases. This underuse likely reflects physician familiarity with VKAs, institutional prescribing habits, and cost-related barriers, all of which contribute to deviation from guideline-recommended therapy.

Taken together, these findings highlight not only clinician-level factors but also system-level barriers—including resource availability, outpatient infrastructure, and institutional routines—that contributed to deviations from guideline-recommended care.

Although our study was designed and conducted before the release of the 2026 AHA/ACC guidelines, several of our observations—particularly regarding echocardiography use, anticoagulation selection, and outpatient management—remain highly relevant in light of the updated recommendations [14].

This study has several limitations. First, its observational design restricted data collection to the in-hospital phase, precluding assessment of long-term outcomes. As with most registry-based studies, diagnostic and treatment decisions were not standardized, relying on individual physician judgment. Variability in the timing of echocardiographic evaluations and biomarker testing may have affected consistency, and sPESI was calculated only at admission, without reassessment during early hospitalization—potentially impacting its prognostic utility. Also, due to the study design, our primary aim was to describe real-world practice patterns and degree of adherence to guidelines rather than to formally investigate the reasons for non-adherence.

Another important limitation is the absence of advanced reperfusion therapies—namely, catheter-directed interventions, surgical embolectomy, and extracorporeal membrane oxygenation (ECMO)—despite their established role in managing high-risk PE. The lack of these options may have influenced outcomes, particularly in patients requiring aggressive intervention. Nevertheless, the study offers valuable real-world insight into PE management practices and highlights areas for improvement in guideline implementation. We also acknowledge that excluding patients who died before diagnostic confirmation or before consent may underestimate early mortality and may reflect delays in diagnostic pathways, representing an unmeasured dimension of guideline adherence.

## Conclusions

This study demonstrates substantial gaps in adherence to guideline-recommended PE management, driven by physician discretion, institutional routines, and socioeconomic constraints. Overuse of D-dimer testing, underuse of V/Q scanning, limited DOAC prescription, and minimal adoption of outpatient pathways highlight key areas for improvement. Strengthening structured care pathways, improving access to recommended therapies, and enhancing clinician familiarity with guideline criteria are essential steps toward optimizing PE outcomes.

## Data Availability

The datasets generated and analyzed during the current study are available from the corresponding author on reasonable request.

## Abbreviations

AC: Anticoagulant
AFib: Atrial Fibrillation
BMI: Body Mass Index
BP: Blood Pressure
BPM: Beat Per Minute
CTPA: Computed Tomography Pulmonary Angiography
DOACs: Direct Oral Anticoagulants
DVT: Deep Venous Thrombosis
ECG: Electrocardiogram/ Electrocardiographic
ECMO: Extracorporeal Membrane Oxygenation
ED: Emergency Department
ESC: European Society of Cardiology
Hb: Hemoglobin
HF: Heart Failure
ICU: Intensive Care Unit
INR: International Normalized Ratio
IVC: Inferior Vena Cava
LL: Lower Limbs
LMWH: Low Molecular Weight Heparin
LVEF: Left Ventricular Ejection Fraction
NSR: Normal Sinus Rhythm
PA: Pulmonary Artery
PASP: Pulmonary Artery Systolic Pressure
PE: Pulmonary Embolism
PESI: Pulmonary Embolism Severity Index
RA: Right Atrium
RBBB: Right Bundle Branch Block
r-tPA: recombinant tissue Plasminogen Activator
RV: Right Ventricle
TAPSE: Tricuspid Annular Plane Systolic Excursion
TR: Tricuspid Regurgitation
TTE: Transthoracic Echocardiography
UFH: Unfractionated Heparin
VKA: Vitamin K Antagonist
VTE: Venous Thromboembolism
